# Scaling‐up biofortified beans high in iron and zinc through the school‐feeding program: A sensory acceptance study with schoolchildren from two departments in southwest Colombia

**DOI:** 10.1002/fsn3.632

**Published:** 2018-04-25

**Authors:** Joni J. S. Beintema, Sonia Gallego‐Castillo, Luis F. Londoño‐Hernandez, José Restrepo‐Manjarres, Elise F. Talsma

**Affiliations:** ^1^ Harvestplus International Center for Tropical Agriculture (CIAT) Cali Colombia; ^2^ Foundation for Agricultural Research and Development (FIDAR) Cali Colombia; ^3^ Division of Human Nutrition Wageningen University and Research Wageningen The Netherlands

**Keywords:** beans, biofortification, Colombia, micronutrients, sensory acceptability

## Abstract

Iron and zinc deficiencies are global health problems, affecting mostly pregnant women and young children. In 2016, biofortified iron and zinc beans were introduced in Colombia. The incorporation of biofortified beans into the national school‐feeding program could facilitate adoption and potentially improve the nutritional status of large populations. However, biofortified beans have to be accepted in order to be consumed by populations. We therefore studied the sensory acceptability of two biofortified beans, BIO‐101 and BIO‐107, and local beans at schools with free feeding services in two departments of southwest Colombia. Measured on a five‐point Likert scale, the mean overall scores were 3.88 ± 0.64, 3.79 ± 0.74, and 3.81 ± 0.76, for BIO‐101, BIO‐107, and the local bean varieties, respectively, without significant differences. The children in Piendamó (Cauca) slightly preferred BIO‐107 over the local bean (*p* < .05) based on color, smell, and taste. The children in Caicedonia (Valle del Cauca) slightly favored the local bean over BIO‐107 (*p* < .05), regarding size, smell, and taste. Overall acceptability in schoolchildren was good for all beans without significant differences. This study advocates incorporation of accepted biofortified beans in the school‐feeding program, in order to reach large groups of schoolchildren and potentially improve their nutritional statuses.

## 1 INTRODUCTION

Worldwide, iron and zinc deficiencies are mayor public health problems and they are often associated with the phenomenon “hidden hunger” (Tulchinsky, 2010). Hidden hunger is a form of malnutrition that occurs when the intake or absorption of micronutrients is too low to maintain a good health and development and is mainly caused by a combination of poor diet, disease, and increased micronutrient needs (Muthayya et al., 2013). In Colombia, mainly pregnant women and very young children are affected by iron deficiency. Zinc deficits mostly occur in preschoolers, especially in indigenous children with more than half of them being zinc deficient (Fonseca et al., 2011). In addition, more children from rural areas suffer from zinc deficiency than children from urban areas (Fonseca et al., 2011).

Food‐based strategies, such as dietary diversification and food fortification, are considered effective approaches to prevent and combat hidden hunger (Bouis, Hotz, McClafferty, Meenakshi, & Pfeiffer, 2011; Verma, 2015). However, their effectiveness largely depends on necessary changes in behavior and diet and could therefore be more difficult to reach (Burchi, Fanzo, & Frison, 2011). Another approach to reduce micronutrient malnutrition that has been demonstrated to be effective is supplementation programs (Imdad, Herzer, Mayo‐Wilson, Yakoob, & Bhutta, 2010), although reaching 100% coverage with these programs proofs to be difficult (Wirth et al., 2017).

A relatively new food‐based strategy to reduce hidden hunger is biofortification, the breeding process that develops and deploys micronutrient‐rich staple crops aiming to improve the nutritional statuses of resource‐poor populations (Bouis et al., 2011; Nestel, Bouis, Meenakshi, & Pfeiffer, 2006). Biofortification is a nutrition‐sensitive agricultural intervention that is relatively inexpensive, cost‐effective, and sustainable considering it involves a single investment in breeding ultimately leading to nutritionally improved crops that will continue to be grown and consumed year after year. By aiming at staple foods, which are predominately present in the diets of poor people, the target population is already familiarized with the crop of interest and this ought to facilitate adoption (Bouis et al., 2011; Nestel et al., 2006). Furthermore, biofortification may be widely accessible, also for people living in remote areas and with limited resources.

Beans (*Phaseolus vulgaris* L.) can be considered important staple crops in the Colombian food basket; according to the national statistics of 2014, Colombians consume on average 3.4 kg per capita of beans yearly (Fenalce, 2015) and 15% of inhabitants eat beans on a daily basis (Alvarez et al., 2005). The common bean is widely affordable, contains substantial amounts of protein, fibers (Arias, Martínez, & Jaramillo, 2007; Geil & Anderson, 1994) and classifies as a source of iron and zinc according to the Codex Alimentarius and the Colombian Ministries of Health and Social Protection (Codex Alimentarius, 1997; INVIMA, 2011; Ministerio de Salud, 1999). Although the concentration of iron in beans is high, bioavailability is influenced by many dietary factors such as food processing techniques, meal composition, the presence of organic acids and antinutrients including polyphenols and phytates (La Frano, Moura, Boy, Lönnerdal, & Burri, 2014). Previously, it was thought that polyphenols are the main inhibitors of successful iron absorption from beans (Beninger et al., 2005). However, Petry et al. (2014) demonstrated that phytic acid is the major inhibitor of iron absorption in beans and that the influence of polyphenols is low when beans are eaten as part of a composite diet.

Biofortification might cause changes in physical and sensory properties of beans, and this could possibly interfere with the consumer acceptability of the newly bred varieties (Nestel et al., 2006). Without acceptation, biofortified beans most likely will not be consumed, obliterating their potential to improve nutritional statuses of vulnerable populations (Birol, Meenakshi, Oparinde, Perez, & Tomlins, 2015).

Two biofortified bean varieties rich in iron and zinc were released in Colombia in 2016.

The national school‐feeding program in Colombia can serve as an important platform for consumer acceptance, by facilitating introduction and familiarization of the biofortified beans among a large, diverse population including the most vulnerable groups (Ministerio de Educación Nacional, 2013). This study will evaluate the sensory acceptability of two biofortified beans against local beans at schools affiliated with the school‐feeding program, in two departments in southwest Colombia.

## 2 MATERIALS AND METHODS

### 2.1 Consent and ethical approval

Ethical approval was obtained from the Institutional Review Board (IRB) of the International Centre for Tropical Agriculture (CIAT) with the IRB case corresponding number 201601‐2. The study protocol was approved by the local education and health secretaries at both study sites and by the school authorities from both educational institutions. The parents or guardians signed the informed consent after explanation of the study procedures. Based on the principles of child's assent, only children who freely participated were included.

### 2.2 Study areas

This study was conducted in southwestern Colombia, in the municipalities Piendamó and Caicedonia in the departments of Cauca and Valle del Cauca, respectively. The sites were chosen because they are situated in areas where the planting and harvest of the biofortified beans take place. Additionally, this study sites are included reflecting geographic differences and somewhat different populations with distinct customs and tastes. Piendamó is a municipality of over 42,000 inhabitants, with predominately rural populations, and is situated in the center of the department of Cauca about 100 km from the city of Cali. The economy is based on agriculture and commerce with the main exports flowers and plantain (DANE, 2006a, 2010).

The municipality of Caicedonia counts over 29,000 inhabitants, with predominately urban populations, and is located in northeast of the department of Valle del Cauca, approximately 172 km from the capital Cali. The economy is based on agriculture and commerce with the main exports of corn, coffee, sugarcane, and plantain (DANE, 2006b, 2010).

### 2.3 Study participants and sampling

Children from sixth‐grade were recruited at educational institutes in Piendamó in November 2016 and in Caicedonia in February 2017. Both institutions are designed for agricultural activities and household students with a stratum 1–2 on a scale from 1–6, indicating low socioeconomic status. Additionally, these institutions were part of the National School Feeding Program, a free feeding service initiated by the government to promote healthy lifestyles and improve learning abilities among schoolchildren (Ministerio de Educación Nacional, 2013). Because the school‐feeding program allows for a semicontrollable situation these populations were chosen.

**Figure 1:**
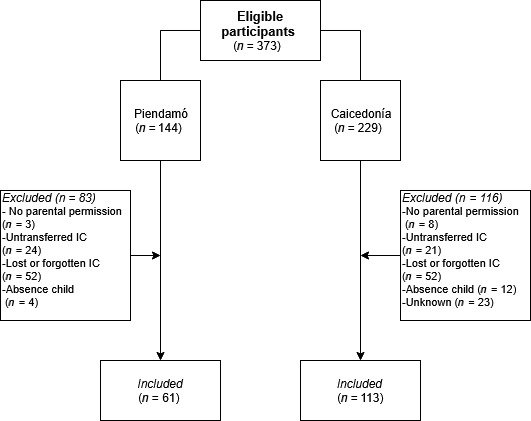
A flowchart describing the inclusion of participants per study site

All sixth‐graders were eligible for participation. Figure 1 shows the flowchart of inclusion of participants by study site. In Piendamó 42% and in Caicedonia 49% of eligible children participated in this study.

### 2.4 Study products and preparation

Two biofortified bean varieties high in iron and zinc named BIO‐101 (ICA, 2016a) and BIO‐107 (ICA, 2016b) were released in Colombia in 2016. These red‐colored bush bean varieties contain on average 8.8 mg/100 g and 8.2 mg/100 g iron and 3.7 mg/100 g zinc and 3.9 mg/100 g zinc, respectively (ICA, 2016a, 2016b). The average iron and zinc concentrations in common bean varieties are estimated to be 5.0 mg/100 g and 3.2 mg/100 g, respectively (Bouis & Welch, 2010). The biofortified varieties are adapted to the Andean region, and they can be grown at altitude levels between 1,200 and 1,800 m. Their returns are considered high with yields between 1.5 and 2 tons, compared with a yield of commercial varieties of 1–1.2 tons. The biofortified beans included in this study were harvested at CIAT, Cali, Colombia in September 2016.

The biofortified samples were evaluated and compared against each other and a local bean variety. This variety was defined as a bush bean that is commonly eaten in the region. In rural, Piendamó Diacol‐Calima was chosen, whereas in urban, Caicedonia Diacol‐Nima was selected as a local control sample. These varieties display similarities in terms of growth pattern, color, growth conditions, and size (Arias et al., 2007). All beans were prepared following the applied recipe in the kitchens of both study sites. About 3 kg of beans per variety was soaked in 12 L of water during 16 hr. After rinsing, the soaked beans were cooked in a cooking pan in 16 L water until softened texture. Cooking times varied between 2 and 2.5 hr, with BIO‐107 being ready‐to‐eat first. During cooking, we added on average 335, 1,460, and 1,000 ml extra water to BIO‐107, BIO‐101, and the local beans, respectively, to prevent dry boiling of the beans. Cooked beans were mixed with a vegetable sauce consisting of tomatoes, onion, and salt. In Piendamó, potato was added to thicken the sauce, whereas in Caicedonia, carrot and green plantain were used to fulfill the same purpose. We assigned the letters A, B, and C to the local bean, BIO‐101, and BIO‐107, respectively to keep the kitchen staff from knowing the differences between the varieties and thus to prevent any different handling.

### 2.5 Study measurements

Study procedures were explained and demonstrated to the children. Prior to the sensory evaluation, participants filled out a short questionnaire to gather information on their age, sex, general liking, and frequency of consumption of beans. Then, sensory evaluations started.

Three small bean samples of 20 g were served all at once in a blinded manner using a neutral tray and cups provided with random assigned three‐digital codes. Each sample set contained a randomly allocated, different serving order to prevent order‐bias. Participants were asked to evaluate the three bean varieties in order on the predetermined attributes color, size, smell, taste, and texture (Calvo & del Rey, 1999; Carrillo Centeno, Chow, Cuadra, Brenes, & Pachón, 2011; Oparinde et al., 2015), and they were instructed to rinse their mouths with water between sample tasting. A five‐point Likert scale, expressed as facial icons, was used to make sure each participant could perform the sensory evaluations individually.

### 2.6 Statistical analyses

All statistical analyses were carried out using IBM SPSS statistics 22 (IBM SPSS Inc., Armonk, NY, USA). Appropriate statistical tests were chosen for ordinal and continuous data.

The overall score per study product, that is, the sum‐up of all sensory attributes measuring the same latent variable on a uniform scale, may be considered continuous data and was therefore assessed using parametric statistics. After normalization of the negatively skewed data by reflected log transformation, paired‐tests were carried out to demonstrate differences between beans within study site. Independent *t* tests were used to show differences in beans across study sites. Findings were reported as mean ± *SD*. The scores of each sensory attribute separately are considered ordinal, as single measures on a five‐point Likert scale cannot give information on relative differences. Therefore, the nonparametric Wilcoxon signed rank test was carried out to assess differences in single attributes between beans within study site. The Mann–Whitney *U* test was conducted to demonstrate differences in sensory attributes of beans across study site. Findings were reported as median values. A *p*‐value <.05 was considered statistically significant.

## 3 RESULTS

Table 1 demonstrates the baseline characteristics of the participants. A total of 174 children participated in this study of which 89 were boys and 85 were girls. Almost twice as many children in Caicedonia participated and these children had a lower median age than their counterparts in Piendamó. An equal percentage of boys and girls participated in both study sites. Generally, more than 90% of children indicated to like beans as opposed to less than 3% of the participants that disliked the crop. Almost 80% of the study population consumes beans on a weekly basis whereas less than 4% rarely or never consumes beans.

**Table 1:** Baseline characteristics of participants by study site

Variables	All (*n* = 174)	Piendamó (*n* = 61)	Caicedonia (*n* = 113)
Age in years (Medium)	11.0	12.0	11.0
Female (%)	48.9	47.5	49.6
General liking beans (%)			
Liked	94.2	93.4	94.6
Disliked	2.3	1.6	2.7
Indifferent	3.5	4.9	2.7
Frequency of bean consumption (%)			
Daily	6.5	6.7	6.4
Weekly	77.6	80.0	76.4
Monthly	13.5	10.0	15.5
Rarely, never	2.4	3.3	1.7

No significant differences (*p *>* *.05) were found between the overall liking scores of the different bean varieties (Table 2). In Piendamó, the children slightly preferred BIO‐107 and BIO‐101 over the local bean (*p *<* *.05), but the overall score between BIO‐101 and BIO‐107 did not significantly differ from each other (*p *>* *.05). The children in Caicedonia slightly favored the local bean and BIO‐101 over BIO‐107 (*p *<* *.05). However, no statistically significant differences in overall hedonic scores were demonstrated between the local bean and BIO‐101.

**Table 2:** Overall mean hedonic scores per study site and bean variety

	All (*n* = 174)	Piendamó (*n* = 61)	Caicedonia (*n* = 113)
Local	3.81 ± 0.76	3.43 ± 0.73^a^	4.02 ± 0.70^b^
BIO‐101	3.87 ± 0.64	3.75 ± 0.57^a^	3.93 ± 0.67^b^
BIO‐107	3.79 ± 0.74	3.84 ± 0.68^a^	3.76 ± 0.77^a^
Differences in mean between study products			
Local versus BIO‐101	−0.06	−0.32*	0.09
Local versus BIO‐107	0.02	−0.41*	0.26*
BIO‐101 versus BIO‐107	−0.08	−0.09	0.17*

Values are given as mean ± *SD*.

Rows reflect mean differences in study samples across setting. Means without a common superscript letter differ *p* < .05.

Columns in the lower part of the table reflect the differences between study samples within study site. Mean differences with a * are statistically significant *p* < .05.

Across study sites, it seemed that the children in Caicedonia liked BIO‐101 and the local bean better than their counterparts in Piendamó (*p *<* *.05). No differences in overall liking of BIO‐107 were found between study sites (*p *>* *.05).

Table 3 shows the median hedonic scores per sensory attribute of each bean variety, with comparisons within and across study sites. Overall, a significant lower median score was given to the size of BIO‐107 (*p *<* *.05) in contrast to the other varieties. Additionally, the smell of BIO‐107 was rated significantly lower than the smell of BIO‐101 (*p* < .05). However, no differences in median bean smell were found between BIO‐107 and the local bean.

**Table 3:** Median hedonic scores of sensory attributes of the local beans, BIO‐101, and BIO‐107 with comparisons within and across study sites

	Bean variety	Bean color	Bean size	Bean smell	Bean taste	Bean texture
All	Local	4.0^a^	4.0^a^	4.0^a,b^	4.0^a^	4.0^a^
	BIO‐101	4.0^a^	4.0^a^	4.0^b^	4.0^a^	4.0^a^
	BIO‐107	4.0^a^	4.0^b^	4.0^a^	4.0^a^	4.0^a^
Piendamó	Local	3.0^a^	4.0^a^	4.0^a^	4.0^a^	3.0^a^
	BIO‐101	4.0^b^	4.0^a^	4.0^a,b^	4.0^b^	4.0^a^
	BIO‐107	4.0^b^	4.0^a^	4.0^b^	4.0^b^	4.0^a^
Caicedonia	Local	4.0^a^	4.0^a^	4.0^a^	4.0^a^	4.0^a^
	BIO‐101	4.0^a^	4.0^a,b^	4.0^a^	4.0^a,b^	4.0^a^
	BIO‐107	4.0^a^	4.0^b^	4.0^b^	4.0^b^	4.0^a^
Differences^a^ Piendamó versus Caicedonia	Local	***p *** **<** *** *** **.001**	*p *=* *.10	***p *** **<** *** *** **.001**	***p *** **<** *** *** **.001**	***p *** **<** *** *** **.001**
	BIO‐101	*p *=* *.27	*p *=* *.91	***p *** **=** *** *** **.03**	*p *=* *.14	*p *=* *.25
	BIO107	*p *=* *.32	*p *=* *.59	*p *=* *.26	*p *=* *.24	*p *=* *.69

Mediums per column without a common superscript letter differ *p* < .05 using Wilcoxon’ s *Z*‐statistic.

a Statistically significant differences between study sites using Mann–Whitney's *U*‐statistic. *p*‐Values in bold indicate significance.

The children in Piendamó gave significantly lower median scores to the local bean variety, in contrast to the other samples, regarding color and taste (*p* < .05). The median smell score of the local bean was also lower rated than the smell of BIO‐107 (*p *<* *.05), but not of that of BIO‐101.

In Caicedonia, children slightly preferred the local bean over BIO‐107 regarding size and taste, by assigning significantly lower median scores to the latter (*p* < .05). Both the local bean and BIO‐101 were given higher median rating scores on smell than BIO‐107 (*p* < .05).

Across setting, the local bean seemed to be rated significantly different regarding color, smell, taste, and texture, with the children in Caicedonia giving the highest median rating scores (*p* < .05). The same children assigned a significantly higher median score to the smell of BIO‐101 than their counterparts in Piendamó (*p* < .05).

## 4 DISCUSSION

We found no differences (*p* > .05) in the overall liking of BIO‐101, BIO‐107, and the local bean varieties as evaluated by 174 sixth‐graders from the departments of Valle del Cauca and Cauca. Although all bean varieties received a rounded overall liking score of 4 of 5 points, the bean size of BIO‐107 was significantly lower rated than BIO‐101 and the local beans. Across study site, the children in Piendamó seemed to like the biofortified beans better than the local bean, mostly due to bean taste and color. In Caicedonia, the children preferred the local bean and BIO‐101 over BIO‐107 because of bean smell. Besides, they liked the size of the local bean better than the size of BIO‐107.

Our results are in line with previous studies on biofortified iron bean varieties, demonstrating either good overall acceptance of biofortified beans rated indifferent or significantly better as opposed to the control beans (Carrillo Centeno et al., 2011; Leyva‐Martínez et al., 2010; Perez, Aparinde, Birol, Gonzalez, & Zeller, 2015; Tofiño et al., 2012). To illustrate, Tofiño et al. (2012) demonstrated similar acceptance patterns of biofortified iron beans against a control bean in Colombian children, although conducted with different beans, preparation form and region in Colombia. Similar to our study, Tofiño and colleagues exposed their participants to beans as part of a dish, in concordance with the usual consumption form. Previous studies have shown that acceptance is context‐dependent and that evaluating food as part of a meal improves acceptability (King, Meiselman, Hottenstein, Work, & Cronk, 2007; King, Weber, Meiselman, & Lv, 2004). This could imply that our findings are a reflection of total meal acceptance rather than the acceptance of beans as separate ingredients and that we can replace commercial beans by more nutritious varieties without opposition as long as other meal components stay the same.

In Colombia, large‐sized beans are preferred among farmers and consumers (Tofiño et al., 2012; Voysest, 2000) and we therefore expected to find differences in bean size liking because of the evidently smaller size of these biofortified beans (23–25 g/100 seeds) as opposed to the local varieties (40–50 g/100 seeds) (Arias et al., 2007). Correspondingly, we demonstrated a significantly lower liking score for the bean size of BIO‐107 as opposed to the local beans; however, no significant differences in bean size were found between BIO‐101 and the local beans. This finding could be explained by the cooking process of BIO‐107 which seemed to result in partial disintegrated (broken) beans. Also in general, the bean size of BIO‐107 was liked less than the size of BIO‐101, despite their approximately equal crude size. Nevertheless, the demonstrated differences in bean size of the varieties did not result in significant different overall liking scores.

Following the principles of central location testing (Lawless & Heymann, 2010), we targeted children from public agricultural institutions affiliated with the national school‐feeding program. This study design was chosen to reach our target population quickly under controlled conditions in a daily setting, while also indirectly targeting other populations such school staff, parents, and agricultural practitioners. By linking biofortified beans to schools through the school‐feeding program, a large group of children could be provided with more nutritious meals that can help improving their nutritional statuses. The involvement of local farmers in crop supply for the school‐feeding program could stimulate local production, such as been done before in home grown school‐feeding projects led by the World Food Program and other partners in different countries (Beltrame et al., 2016; Masset & Gelli, 2013).

It has been previously shown that nutritional campaigns providing nutritional information enhance the acceptability and demand of biofortified iron beans (Murekezi, Oparinde, & Birol, 2017; Oparinde et al., 2015; Perez et al., 2015). Therefore, the integration of the school‐feeding program through nutritional education in the curriculum of schools could potentially facilitate acceptance of biofortified beans and benefit scaling‐up strategies, among the diverse population connected to the school.

A recent randomized controlled trial among Rwandan women demonstrated significantly improved iron statuses after consuming biofortified iron beans as part of a plant‐based diet for duration of 128 days, supporting the efficacy of the intervention (Haas et al., 2016). In addition, improved cognitive performance was demonstrated in the women who had consumed biofortified iron beans (Murray‐Kolb et al., 2017). These women, however, consumed 300–350 g beans daily under ideal feeding conditions, while the majority of our population eats beans on a weekly basis. In addition, depending on the modality of the school, the school‐feeding program offers to the utmost 45 g of legumes including beans three times per week (Ministerio de Educación Nacional, 2013). That being said, our population most likely absorbs nonhaem iron from beans more efficient due to frequent consumption of meat and chicken at school (Abbaspour, Hurrell, & Kelishadi, 2014). Nevertheless, biofortified beans should not be deployed as a single intervention but as part of a multi‐food approach including various biofortified crops and nonstaple foods in order to impact the nutritional statuses of schoolchildren through the school‐feeding program.

### 4.1 Strengths and limitations

The fact that this study was held under actual conditions in a day‐to‐day setting with frequent product‐users and local recipes can be considered strengths of the current research. Besides, the chosen statistics are worth mentioning as the analysis of an ordinal scale is often misused (Jamieson, 2004). In many cases, mean difference testing of single Likert‐items is more a function of sample size than respondent attitude, effectuating misleading results and incorrect conclusions (Clason & Dormody, 1994).

However, this study also had some limitations. First of all, we intended to include all sixth‐graders of both institutions. Although, more than half of the eligible students at both study sites were excluded from participation, possibly leading to nonresponse bias (Berg, 2005). Yet, child's main reasons (Figure 1) for not participating had mostly to do with forgetfulness and disorganization, emotional traits considered perfectly normal during middle childhood (EXTE U, 2011). The potential impact of the nonresponse bias is therefore expected to be small or nonpresent.

Secondly, almost twice as many students from Caicedonia than from Piendamó took part in this study, making the results of the former more substantial. Theoretically, equal absolute contributions of both study sites could have led to a higher overall score of BIO‐107 as opposed to the other varieties, as the minority group in Piendamó had a clear preference for this variety.

A third limitation would be the use of different local bean varieties as controls. Because the control bean should be a variety familiar to the population, the inclusion of two different kinds is nearly inevitable, due to differences in customs across sites. Although we included highly equivalent local bean varieties, we did find differences in liking between them, inducing disproportional liking of the biofortified beans against the local beans across study site. However, these differences cannot be considered exact, as different participants carried out the evaluations.

A fourth more methodologically issue could have been the use of a five‐point Likert scale over the nine‐point Likert scale. In general, the 9‐point scale is preferred because it provides more alternatives with approximately equal subjective spacing, justifying the use of more powerful statistics (Lim, 2011). Although the use of the 9‐point scale has been established in children the same age of our participants (Kroll, 1990), the five‐point version is often preferred because diversity in skill level can interfere with individual performance of the evaluations (ASTM, 2013). In addition, the study of Weijters, Cabooter, & Schillewaert (2010) supports the use of a five‐point Likert scale within general public respondents regardless of age, as individual task performance is a matter of cognitive ability rather than maturity. This explanation also supports our choice to include sixth‐graders in this study rather than the actual targeted preschoolers for micronutrient intervention. Elaborating further on the relevancy of chosen study population, it is important to state that although low prevalence of iron deficiency is reported among school children (Fonseca et al., 2011), prevalence substantially increases in adolescence (Fonseca et al., 2011). This increase has likely to do with insufficient compensation of higher iron needs and in case of girls higher iron loses. Considering schoolchildren are at preadolescent stage, it seems commendable to yet start intervention in order to create optimal circumstances to meet future demands.

A final discussion point relates to the generalizability of our findings. One could question the applicability of our results, as the current study was conducted in a specific population group and setting. Nevertheless, we expect similar results in other population groups in Colombia in terms of age, socioeconomic background, and habitat, as biofortified beans are generally well accepted and that liking can be positively influenced by nutrition information and possibly by context as well.

## 5 CONCLUSION

The overall acceptability in schoolchildren was good for all bean varieties without significant differences. Although the bean size of BIO‐107 was significantly lower rated than that of BIO‐101 and the local beans, this did not generate significant differences in overall liking between varieties. These findings suggest that our biofortified beans can be successfully implemented, and potentially help reducing iron and zinc deficiencies in school children. However, as the bean consumption of Colombian children is most likely insufficient to generate a substantial effect on iron and zinc status, biofortified beans should be offered as part of a multi‐food approach in order to have a meaningful effect, while also addressing other micronutrient deficiencies. The school‐feeding program lends itself perfectly for multi‐food strategies and the uptake of biofortified beans has the ability to improve existing programs with the aim of promoting healthy lifestyles and learning abilities among children.

Future acceptance studies should investigate whether a combination of different biofortified crops (maize, rice, beans, sweet potato) can be incorporated successfully in the school‐feeding program, followed by efficacy studies to determine the impact of different biofortified crops on the nutritional status of school populations as part of a multi‐food‐based approach.
